# Body size and temperature affect metabolic and cardiac thermal tolerance in fish

**DOI:** 10.1038/s41598-023-44574-w

**Published:** 2023-10-19

**Authors:** Krista Kraskura, Emily A. Hardison, Erika J. Eliason

**Affiliations:** Department of Ecology, Evolution and Marine Biology, University of California, Santa Barbara, CA 93106 USA

**Keywords:** Ecophysiology, Animal physiology

## Abstract

Environmental warming is associated with reductions in ectotherm body sizes, suggesting that larger individuals may be more vulnerable to climate change. The mechanisms driving size-specific vulnerability to temperature are unknown but are required to finetune predictions of fisheries productivity and size-structure community responses to climate change. We explored the potential metabolic and cardiac mechanisms underlying these body size vulnerability trends in a eurythermal fish, barred surfperch. We acutely exposed surfperch across a large size range (5–700 g) to four ecologically relevant temperatures (16 °C, 12 °C, 20 °C, and 22 °C) and subsequently, measured their metabolic capacity (absolute and factorial aerobic scopes, maximum and resting metabolic rates; AAS, FAS, MMR, RMR). Additionally, we estimated the fish’s cardiac thermal tolerance by measuring their maximum heart rates (*f*_Hmax_) across acutely increasing temperatures. Barred surfperch had parallel hypoallometric scaling of MMR and RMR (exponent 0.81) and a weaker hypoallometric scaling of *f*_Hmax_ (exponent − 0.05) across all test temperatures. In contrast to our predictions, the fish’s aerobic capacity was maintained across sizes and acute temperatures, and larger fish had greater cardiac thermal tolerance than smaller fish. These results demonstrate that thermal performance may be limited by different physiological constraints depending on the size of the animal and species of interest.

## Introduction

Several ectothermic species are decreasing in body size directly in response to environmental warming^1–5^. This is especially concerning in fisheries species because of their importance in providing food security, sustaining economies, and supporting human well-being through recreational opportunities^6^. Within a given fish species, vulnerability to temperature change is often highest during early life stages and in spawning adults^4,7–9^. However, the physiological mechanisms underpinning these trends remain unresolved. One hypothesis suggests that there is a temperature- and size-specific mismatch between a fish’s rising metabolic demand (metabolic rate, MR) and its ability to supply oxygen to meet this demand (e.g., cardiorespiratory mechanisms; diffusion of O_2_ across the gills, heart rates, *f*_H_)^10,11^. Specifically, the loss of cardiac function is closely linked with the decline in metabolic capacity and thermal tolerance in numerous ectotherms^12–17^. The heart supports aerobic capacity in fishes by delivering oxygen, nutrients, and hormones to the working tissues and by removing metabolic waste^14,18,19^. Therefore, cardiac function may be the mechanism driving size-specific vulnerability to warming in fishes.

**Figure 1:**
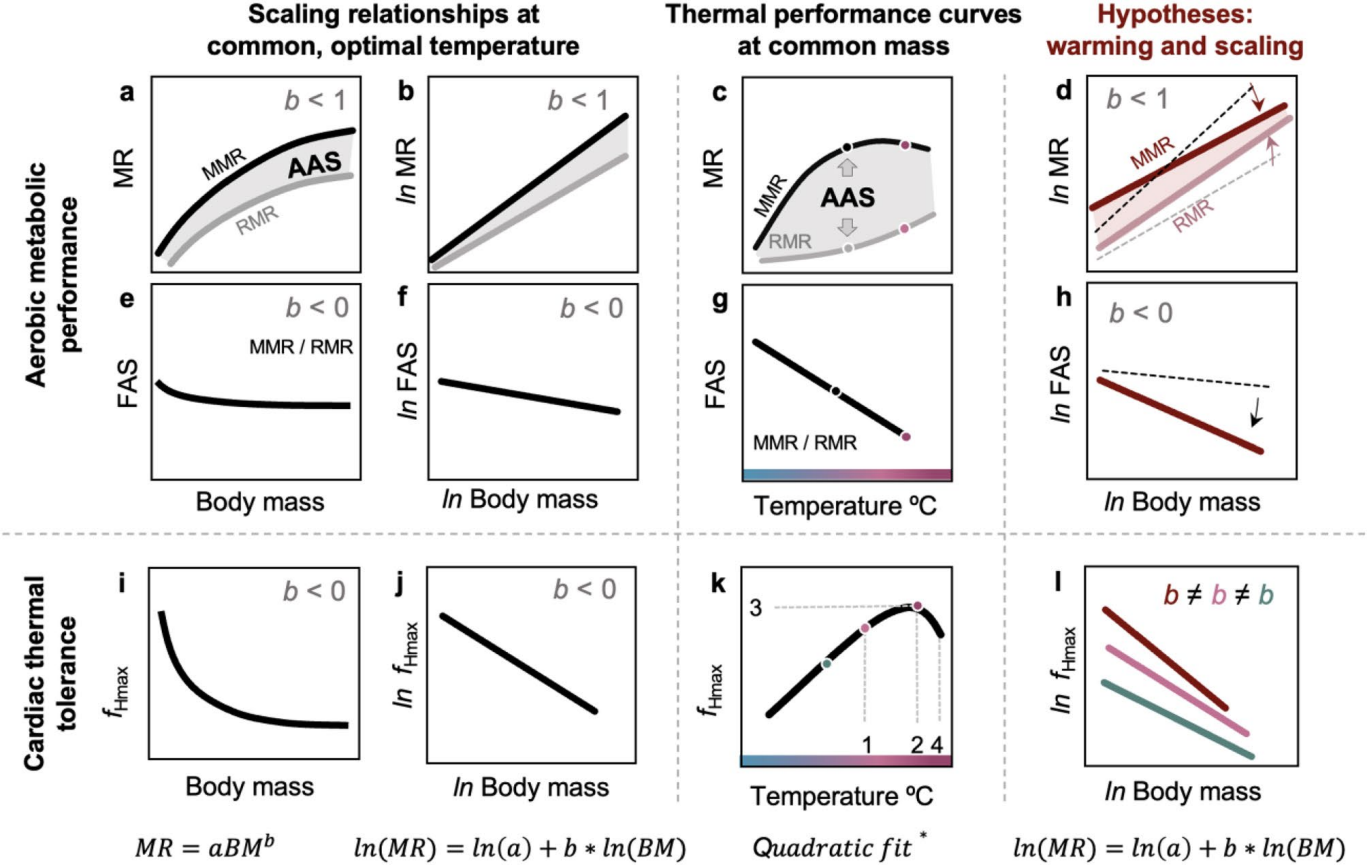
Conceptual presentation of body size and temperature influence on aerobic metabolic rate, maximum heart rate, and cardiac thermal tolerance in ectotherms. (**a**,**b**) Scaling of maximum and resting metabolic rates (MMR and RMR, dark and light lines, respectively), and absolute aerobic scope (AAS = MMR–RMR; shaded) under optimal thermal conditions. (**e**,**f**) Scaling of factorial aerobic scope (FAS = MMR/RMR), a metric indicating metabolic constraint under optimal temperatures. (**i**,**j**) Scaling of maximum heart rates (*f*_Hmax_). Individual or species-specific thermal performance curves without considering body size are depicted for MMR, RMR, AAS (**c**), FAS (**g**), and *f*_Hmax_ (**k**). (**d**,**h**,**l**) The hypothesized scaling relationships under temperatures above optimal for the animal. Directional change in scaling from optimal temperatures (greyscale, dashed lines) to warm are presented in panels (**d**,**h**). The equations for each column are provided on the bottom; BM = body mass, *a* = scaling intercept, *b* = scaling slope; * the quadratic fit = 2nd order polynomial fits can be used to describe TPC. Isometric scaling (*b* = 1 or −1) describes proportional change in performance with body mass. No scaling (*b* = 0) describes mass independence. The dots in (**c**,**j**,**k**) mark temperatures presented in panels (**d**,**h**,**l**) respectively. Cardiac thermal tolerance metrics are shown in (**k)**: 1 = T_AB_, breakpoint temperature; 2 = T_PEAK,_ the temperature at peak *f*_Hmax_; 3 = PEAK_*f*Hmax_ corresponding to T_PEAK_; 4 = T_ARR_, the temperature at first cardiac arrhythmia. All figures are for conceptual representations, only.

Scaling relationships describe how body size affects any trait, including MR and *f*_H_^20^. Metabolic rates commonly scale with body mass following a positive power function, MR = a*BM^*b*^, where *b* < 1 when estimated across-taxa^21^ and tend to range from *b* = 0.80 to *b* = 0.89 in fishes^21–25^ (BM = body mass, *a* = a context-specific coefficient, *b* = scaling slope or exponent; Fig. 1). However, scaling slopes and intercepts vary inter-specifically and intra-specifically^24–26^, with activity level^11,22^, across species lifestyles, and in response to temperature^22,28,30,31^. Many researchers have explored how these various factors alter the scaling of MR, including temperature^11,23,27,32,33^, but the mass-scaling of cardiac thermal tolerance and heart rates in ectotherms remains unclear. Unlike metabolism, heart rates tend to scale negatively with body mass^34^ (e.g., cockroaches^35^, cetaceans^36^, snakes^37^). Specifically, it is proposed that scaling of *f*_H_ follows a reciprocal function to metabolism, *f*_H_ = a*BM^−*b*^ (e.g., *b ≈* − 0.25 mammals and birds^20,38–40^), but in fishes, the relationship between *f*_H_ and body mass can be bell-shaped^35^ and flat^18,41^ (*b* = 0).

Besides body size, temperature is the most prominent factor governing physiological rates in ectotherms^42,43^. The temperature dependence of biological rates is described by thermal performance curves, TPC^44^, which are performance-specific and typically non-linear (Fig. 1)^45–47^. Resting metabolic rates (RMR) measured in resting, non-reproducing, non-digesting ectotherms rise exponentially with increasing temperature (Fig. 1c). In contrast, maximum metabolic rate (MMR) may rise continuously with increasing temperature, peak and plateau, or peak and decline^44^ (Fig. 1c). Factorial aerobic scope (FAS = MMR / RMR) generally declines with increasing temperatures^48^ (Fig. 1g), which indicates an increasing metabolic constraint with warming. Alternatively, absolute aerobic scope (AAS = MMR–RMR), which represents an individual’s aerobic capacity to thrive (e.g., move, digest, reproduce), peaks at optimal temperatures and may plummet towards both warm and cold temperatures (Fig. 1a). Similarly, maximum *f*_H_ (*f*_Hmax_) increases steadily with warming until it first begins to slow at a breakpoint temperature (T_AB_) and then *f*_Hmax_ reaches the peak (PEAK*f*_Hmax_ at the corresponding peak temperature, T_PEAK_) (Fig. 1k). The temperature after T_PEAK_ when the heartbeat becomes irregular (arrhythmic) is T_ARR_ (°C) (Fig. 1)^12^. T_AB_, T_PEAK_ and T_ARR_ provide key functional temperature tolerance indices derived from TPCs of *f*_Hmax_ and are directly linked with aerobic metabolic capacity^12,49,50^ (Fig. 1k). TPCs for metabolic rates can be life stage and thus body size specific^51,52^ suggesting that TPCs of *f*_Hmax_ and cardiac thermal tolerance could also change with body size. Therefore, the scaling of metabolic rates and cardiac thermal tolerance may differ across temperatures.

**Figure 2:**
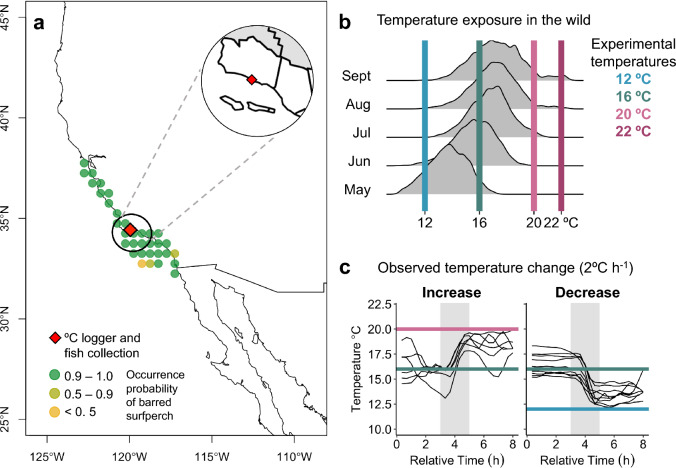
Habitat range and thermal conditions of barred surfperch. (**a**) Pacific coastline in North America with an inset of Santa Barbara and Ventura Counties (white) in California, U.S. Red diamond marks Naples Kelp Forest, a temperature monitoring site (data in **b** and **c**; Santa Barbara Coastal Long Term Ecological Research program^107^; coord: 34.42388, −119.95053), and fish collection site (Haskell’s Beach; 34.430767, −119.916717). Collection site was approximately 2 km from the temperature logger. (**b**) Recorded temperatures from May to September across years 2001–2021 with marked ecologically relevant temperatures (12, 16, 20, 22 °C). (**c**) Recorded temperatures across 8 h presenting examples for selected acute temperature change treatments (shaded grey: temperatures change from 16 °C at a green line to 20 °C (n = 9; pink line) and to 12 °C (n = 9; blue line) at an average rate of 2 °C h^−1^). Native range sourced from fishbase.org, referencing Global Biodiversity Information Facility (https://www.gbif.org/) and Ocean Biodiversity Information System (https://obis.org/).

Here, we studied the metabolic rates and cardiac thermal tolerance of barred surfperch (*Amphistichus argenteus*), a temperate viviparous marine fish species from a thermally dynamic coastal habitat (surf zone). Coastal temperatures change seasonally (Fig. 2b) but are also characterized by high daily thermal variability (Fig. 2c). To thrive in the surf zone, barred surfperch must be able to respond to the acute temperature swings^44,53–56^ they frequently encounter. Additionally, barred surfperch are a good model for studying size and life stage-specific physiology because they give live birth to fully developed juveniles (< 3 g, lab-measured), reach an adult size of ~ 2.0 kg^57^, and live in the surf zone their entire lifetime, thus juveniles, subadults, and spawning adults experience the same thermal conditions. We measured each individual’s metabolic capacity across acute ecologically relevant temperatures (12, 16 (control), 20, 22 °C; Fig. 2), and *f*_Hmax_ during acute warming from 16 °C to the upper functional temperature limit, or where the heart became arrhythmic. We hypothesized that the perch’s MMR and RMR would differ in their scaling slopes and in response to acute temperature change^23,58,59^. Specifically, we predicted that (i) *b*_MMR_ > *b*_RMR_ under optimal temperature conditions^59–61^ (Fig. 1b), that (ii) *b*_MMR_ would decrease with increasing temperatures because larger individuals may have compromised cardiovascular and oxygen supply capacity compared to smaller counterparts^11,62,63^ and (iii) *b*_RMR_ would increase with increasing temperatures because larger mature individuals are more temperature sensitive and likely invest more energy than small individuals towards reproduction when food is not limited (Fig. 1d)^64^, thus (iv) *b*_AAS_ < *b*_MMR_ < *b*_RMR_ and *b*_FAS_ < 0 under warming (Fig. 1h). These trends would reveal a decline in aerobic performance in larger adult fish under warming^11,65^. Further, we hypothesized that *f*_Hmax_ would scale negatively^4^, *b*_*f*Hmax_ < 0, and that *b*_*f*Hmax_ would decrease with increasing temperature, the rationale being that PEAK*f*_Hmax_ would be lower in larger fish (Fig. 1l). Our study provides mechanistic insight into temperature-modulated mass scaling relationships of aerobic capacity, and cardiac thermal tolerance in fishes.

## Results

### Scaling relationships

**Figure 3:**
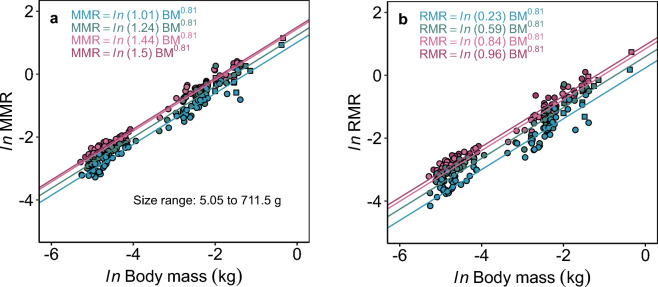
Consistent hypoallometric scaling of maximum and resting metabolic rates across acute temperatures in barred surfperch. (**a**) Maximum metabolic rates (MMR) and (**b**) resting metabolic rates (RMR) scaled similarly with *b* = 0.81 and consistently with acute temperature change from 16 °C to 12 °C (blue), 16 °C (green; control), 20 °C (light pink), and 22 °C (dark pink) (mixed model estimates). The squares are reproductively active females. Fish were repeat tested at each temperature; MMR: n = 83 (238); RMR: n = 81 (233), individuals (observations).

Aerobic metabolic performances (MMR, RMR, AAS) scaled hypoallometrically with body size, and the acute temperature change consistently impacted the scaling intercepts but not the slopes (*b*_AAS_ > *b*_MMR_ ≈ *b*_RMR_) (Fig. 3). This suggests that body size and temperature independently, not interactively, affect aerobic metabolism in barred surfperch (models with the interaction between temperature and mass were ∆BIC > 15; Supplementary Table S1 online). Specifically, MMR scaled with *b* = 0.810 {CI_95%_: 0.79, 0.83} with significantly increasing intercepts across increasing temperature (ANOVA: *ln*(a): χ^2^_(3)_ = 463.88, P < 2.2e−16) (Fig. 3a). The scaling slope of RMR was consistent at *b*_RMR_ = 0.809 {CI_95%_: 0.77, 0.85}, also with significantly increasing intercepts with increasing temperatures (ANOVA: *ln*(a): χ^2^_(3)_ = 515.971, P < 2.2e−16) (Fig. 3b).

Further, the scaling of aerobic scope (both AAS and FAS) did not indicate that larger barred surfperch had a lower aerobic metabolic performance than the smaller perch. AAS scaled hypoallometrically and consistently across temperatures with *b*_AAS_ = 0.883 {CI_95%_: 0.85, 0.92} (Table 1). Opposite to our prediction, FAS scaled weakly and positively across temperatures with *b*_FAS_ = 0.020 {CI_95%_: − 0.01, 0.05} (Table 1). Additionally, all temperature-specific scaling relationships of AAS were *b*_AAS_ ~ 0.88 (Supplementary Fig. S1, Table S2 online). Together, the resulting *b*_AAS_ > *b*_RMR_ ≈ *b*_MMR_ suggests that larger fish did not lose aerobic capacity with acute warming. Similarly, the positive scaling of FAS suggests that larger individuals did not develop a greater aerobic constraint under acute warming compared to smaller individuals.

**Table 1:** Mass scaling relationships of measured physiological performances.

Physiological performance	n indiv. (n obs.)	T (°C)	Scaling slope, *b* (SE) {CI_2.5%,_ CI_97.5%_}	Intercept, *ln*(*a*) (SE)
MMR (mgO_2_ min^−1^)	83 (238)	12	0.810 (0.010) {0.79, 0.83}	1.011 (0.039)
		16		1.235 (0.022)
		20		1.436 (0.022)
		22		1.497 (0.03)
RMR (mgO_2_ min^−1^)	81 (233)	12	0.809 (0.020) {0.77, 0.85}	0.235 (0.063)
		16		0.59 (0.03)
		20		0.845 (0.031)
		22		0.97 (0.042)
AAS (mgO_2_ min^−1^)	81 (233)	12	0.883 (0.019) {0.85, 0.92}	0.541 (0.075)
		16		0.591 (0.047)
		20		0.745 (0.048)
		22		0.713 (0.065)
FAS	81 (233)	12	0.020 (0.016) {-0.01, 0.05}	0.819 (0.049)
		16		0.685 (0.025)
		20		0.637 (0.026)
		22		0.571 (0.035)
*f*_Hmax_ (beats min^−1^)	30 (317)	16*	-0.052 (0.009) {-0.07, -0.03}	4.325 (0.034)
T_ARR_ (°C)	29		0.030 (0.010) {0.01, 0.05}	3.359 (0.037)
T_PEAK_ (°C)	30		0.034 (0.012) {0.01, 0.06}	3.304 (0.041)
PEAK_*f*Hmax_ (beats min−1)	30		*-*	4.916 (0.018)
T_AB_ (°C)	27		0.030 (0.010) {0.01, 0.05}	3.144 (0.033)
Ventricle Mass (kg)	30		0.855 (0.029) {0.80, 0.91}	-7.639 (0.097)

MMR, RMR, AAS, FAS, and *f*_Hmax_ scaling relationships were estimated using mixed models with temperature (T) as categorical explanatory variable. Cardiac thermal tolerance metrics and ventricle size were estimated using simple linear regressions. SE = standard error of mean.

*Maximum heart rates changed significantly with 1 °C incremental change in temperature, only the scaling intercept for 16 °C estimate are presented.

**Figure 4:**
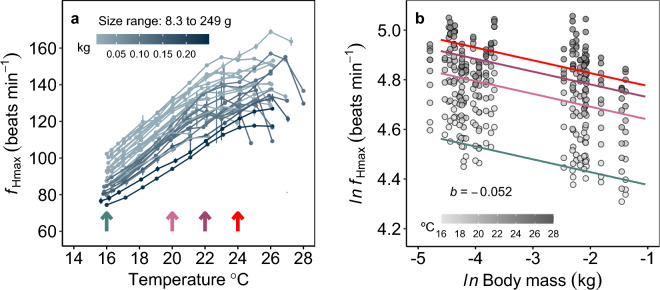
Negative and consistent scaling of maximum heart rate (*f*_Hmax_) in barred surfperch across acute temperatures. (**a**) Individual *f*_Hmax_ across acutely increased temperatures; the errors are SD of *f*_H_ across 15 s. (**b**) Estimated common mass scaling relationships of *f*_Hmax_ across all temperatures (mixed model). In all panels: colored lines and symbols indicate 16, 20, 22 °C where metabolic rates were measured in the same fish, and mortality was observed following acute exposure to 24 °C (red markers). n = 30 all panels.

The *f*_Hmax_ scaled consistently negatively with body mass with *b*_*f*Hmax_ = − 0.052 {CI: − 0.07, − 0.03} (Fig. 4). Therefore, body mass and temperature independently influenced *f*_Hmax_ (models with T * *ln*(BM) were ∆BIC > 45) (Fig. 4b). At their acclimation temperature (16 °C), the fish with the lowest recorded *f*_Hmax_ (74.40 beats min^−1^) was 233.0 g, and the fish with the highest recorded *f*_Hmax_ (104.38 beats min^−1^) was 12.59 g (Fig. 4a). The scaling slope of *b*_*f*Hmax_ decreased at higher acute temperatures (*b* = − 0.068 at 16 °C to *b* = − 0.036 at 24 °C; Supplementary Fig. S2, Table 1 online).

**Figure 5:**
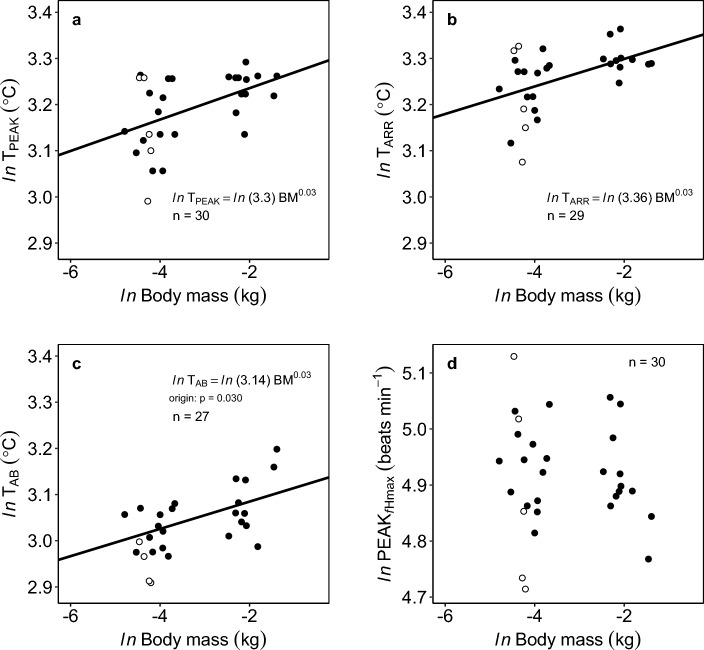
Mass scaling of cardiac thermal tolerance indices. The T_PEAK_ (**a**)_,_ T_ARR_ (**b**), and T_AB_ (**c**) were weakly but significantly associated with body mass, but PEAK_*f*Hmax_ (**d**) was independent of mass. In all panels: open symbols show laboratory-born juveniles and closed symbols are wild-collected fish (origin). Size range: 8.3 to 249 g.

The TPCs of *f*_Hmax_ varied across individuals of different sizes (Fig. 4a). All cardiac thermal tolerance metrics, T_ARR_, T_AB_, and T_PEAK,_ similarly and positively scaled with body mass (*b* = 0.030 to 0.034; Fig. 5a,b,c; Supplementary Fig. S3 online). The T_AB_, T_PEAK_, and T_ARR_ were lower in juvenile perch (< 50 g) compared to the adults by an average of 1.62 °C, 1.79 °C, and 1.72 °C, respectively. Specifically, the smaller wild-caught fish (~ 10 g) had a ~ 1.5–2 °C lower T_AB_, T_ARR_ and T_PEAK_ compared to the larger fish (> 100 g). The T_PEAK_ ranged between 19.90–26.90 °C (n = 30, size range: 8.3–249 g), while the cardiac arrhythmias (T_ARR_) ranged between 21.66 and 28.90 °C (n = 29). At an individual level, the measured values of two upper thermal tolerance metrics, T_PEAK_ and T_ARR_, were only apart by a mean of 1.75 °C (range: 0.47–3.70 °C; T_ARR_ *minus* T_PEAK_). The T_AB_ had a broad range: 18.34 °C (14.90 g fish) and 24.49 °C (249 g fish), but was significantly lower in laboratory-born juveniles compared to wild-caught juveniles (ANOVA: F_(1)_ = 5.28, P = 0.031) (Supplementary Table S3 online). The PEAK_*f*Hmax_ was not significantly associated with body mass (Fig. 5d, Supplementary Table S3 online) but did not exceed 168.9 beats min^−1^ across tested fish. Lastly, ventricle mass scaled with body mass with *b* = 0.855 {CI_95%_: 0.80, 0.91} (Table 1, Supplementary Fig. S4 online). Altogether, we found that larger fish had slightly higher cardiac thermal tolerance compared to juveniles.

### Mass-independent temperature effects

**Figure 6:**
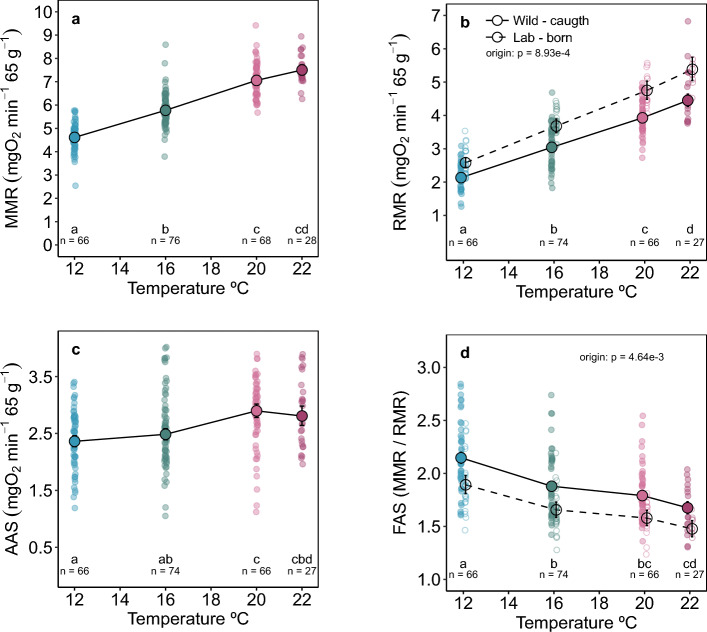
Metabolic performance is maintained across a broad range of acute temperatures (12, 16, 20, 22 °C) in barred surfperch. Mass-specific and mass-normalized (65 g fish, mean size in the study) aerobic metabolic performance across temperatures: (**a**) Maximum metabolic rate (MMR), (**b**) Resting metabolic rates (RMR), (**c**) Absolute aerobic scope (AAS = MMR–RMR), and (**d**) Factorial aerobic scope (FAS = MMR /RMR) (**d**). Shaded symbols are individual fish, the large, solid symbols are estimated group means (± SE). Origin refers to lab-born versus wild-caught juveniles. Fish were repeat tested across temperatures; MMR: n = 83 (238); RMR: n = 81 (233), individuals (observations); Table 1.

Mass-corrected and mass-specific MMR and RMR increased with increasing acute temperatures between 12 to 20 °C. The fish’s mean MMR increased from 4.61 mgO_2_ min^−1^ 65 g^−1^ at 12 °C to 7.50 mgO_2_ min^−1^ 65 g^−1^ at 22 °C, but significantly changed only up to 20 °C (wild-caught fish; post-hoc: 12 vs. 16 °C, 16 vs. 20 °C: P < 0.001, Supplementary Tables S3, S4 online) (Fig. 6a), and their RMR increased significantly across all acute temperatures (mean at 12 °C: 2.133 mgO_2_ min^−1^ 65 g^−1^, at 22 °C: 4.45 mgO_2_ min^−1^ 65 g^−1^; wild-caught fish) (Fig. 6b, Supplementary Tables S3, S4 online). Additionally, laboratory-born juveniles had significantly higher RMR than wild-caught juveniles (ANOVA: χ^2^_(1)_ = 11.035, P = 8.94e−4).

The similar increase of MMR and RMR with acute warming resulted in minimal change in AAS across temperatures. The highest estimated mean was 2.90 mgO_2_ min^−1^ 65 g^−1^ at 20 °C (mass-normalized to 65 g; Fig. 6c) (post-hoc: 16 vs. 20 °C P = 0.0076; 12 *vs.*16 °C and 20 vs. 22 °C, P = ns). These findings suggest that aerobic metabolic capacity in barred surfperch was maintained across acute ecologically relevant acute temperatures.

In contrast, FAS declined with increasing temperature. The FAS was the highest at 12 °C (mean = 2.08; range = 1.27–3.60), and it dropped to its lowest at 22 °C (mean = 1.57; range = 1.29–2.17) (Fig. 6d). Similarly to RMR, FAS was significantly lower in laboratory-born juveniles (ANOVA: χ^2^_(1)_ = 8.013, P = 0.005; Supplementary Table S3 online). Therefore, the fish of all sizes were experiencing an aerobic metabolic constraint that increased with acute warming.

**Figure 7:**
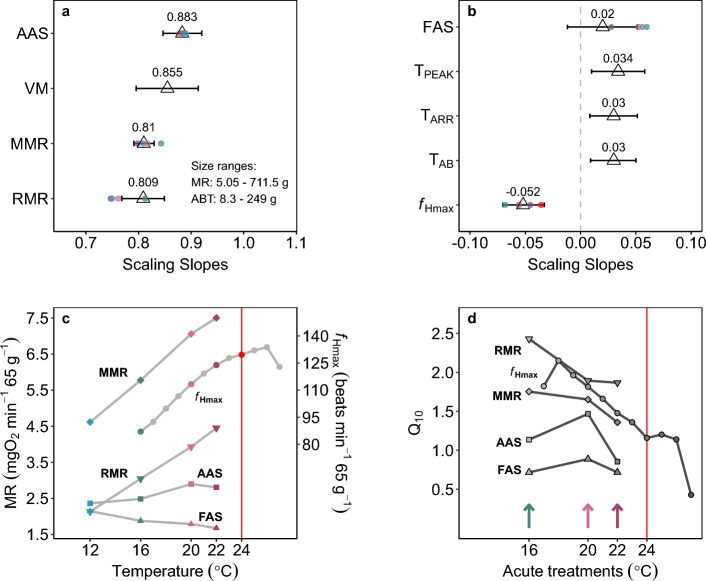
Scaling relationships and acute thermal performance across physiological metrics in barred surfperch. (**a,b**) Scaling slopes of metabolic and cardiac performance were not consistently different across acute temperatures but varied across traits (white triangles; slope values noted above, error is a 95% CI; mixed model results). Colored circles are temperature-specific scaling slopes. Thermal performance curves (**c**) and Q_10_ (**d**) of mean mass-independent aerobic metabolic performances and *f*_Hmax_. Red symbol and line mark 24 °C, a temperature at which 50% mortality was observed during a respirometry trial. MR = metabolic rate (RMR = resting; MMR = maximum), AAS = absolute aerobic scope, FAS = factorial aerobic scope, T_ARR_ = temperature at first cardiac arrhythmia, T_AB_ = Arrhenius breakpoint temperature, T_PEAK_ = temperature at highest recorded *f*_Hmax_, VM = ventricular mass.

The temperature sensitivity of mass-independent aerobic and cardiac performances was generally low (Q_10_ ≤ 2) and decreased with increasing temperatures. The RMR was the most temperature-sensitive, followed by *f*_Hmax_, and MMR (Fig. 7). MMR and RMR increased with increasing temperature, but above 20 °C, the rate of increase slowed (Figs. 6a,b, and 7d). The RMR increased most rapidly from 12 to 16 °C (Q_10_ (12–16 °C) = 2.43, Q_10_ (16–20 °C) = 1.89, and Q_10_ (20–22 °C) = 1.87, Fig. 7d). Meanwhile, the Q_10_ of MMR remained low across all test temperatures (Q_10_ < 1.75, Fig. 7d). The Q_10_ of *f*_Hmax_ decreased steadily across temperatures from ~ 2.0 to 1.35 and plateaued after ~ 23 °C (Q_10_ ~ 1.20) until a precipitous drop at 27 °C (Fig. 7d). The fish’s aerobic scopes (AAS and FAS) were the least temperature-sensitive. Specifically, the Q_10_ values for AAS were below 1.46, and Q_10_ for FAS were all < 1.0 (Fig. 7d). Lastly, considerable interindividual variation was common across all performances and temperatures (Supplementary Table S4 online).

## Discussion

Several fish species have been declining in body mass^1,66^, suggesting that larger individuals may be more vulnerable to warming than smaller ones^2^. However, the underlying mechanisms of this intraspecific trend are unclear^2,53,64^. If warming causes metabolic scaling slopes to shift such that *b*_MMR_ < *b*_RMR_ (Fig. 1), then it would indicate a proportionally greater loss of aerobic capacity with increasing temperatures in larger individuals^11^. Therefore, measuring species-specific scaling relationships across ecologically-relevant temperatures and thermal exposure times can help us identify how vulnerability may change across a fish’s lifetime.

Here, the consistent and parallel *b*_MMR_ and *b*_RMR_ (*b* = 0.81) across a 10 °C temperature range (12–22 °C) suggests that aerobic capacity was not suffering under acute warming in larger barred surfperch. This was reaffirmed by the consistent, slightly positive *b*_FAS_ = 0.02 (*b*_FAS_ ≈ 0.05 temperature-specific) and the consistent *b*_AAS_ = 0.88 (Supplementary Table S2 online). The temperature-insensitive *b*_MMR_ ≈ *b*_RMR_ may be explained by the distinctive lifestyles and life history of barred surfperch. The temperature sensitivity of metabolic scaling is known to be species-specific^23,27,32,33^. For example, after a 2–4 week temperature-acclimation, *b*_RMR_ decreased with increasing temperature in round stingray^67^ and coregonids^33^, did not change in cyprinids^33,68,69^, and varied in response to changing temperatures in Atlantic cod^70^, European perch^71^, and yellow perch^72^. However, interspecific level meta-analyses on fish suggest that scaling slopes decrease with increasing temperatures^11^. Interspecific variation in how temperature affects RMR and MMR scaling relationships could be caused by various factors, including species ecology^22^, acclimation^71^, phylogeny^33^, and intraspecific reproductive trade-offs^31,62^, and possibly the methodology used to measure metabolic rate (i.e., especially MMR,^73–76^). For perch, they spend their entire lifetime in the surf zone, where they routinely maneuver into the swash zone to feed on hard-shelled sand crabs^77^. They also experience rapid, acute (hourly), as well as longer-term (seasonal) temperature variations (Fig. 2), which likely accounts for the low acute thermal sensitivity of metabolic rate across a broad range of temperatures. Therefore, their ecology and generally non-athletic lifestyles (minimal difference between MMR and RMR) could partly explain why scaling of RMR and MMR was similar and insensitive to acute temperature change (e.g., metabolic level boundary hypothesis^59,60^). Barred surfperch may be living close to their full aerobic capacity investing most of their available energy in foraging, digestion, growth, and reproduction, though prioritization of these different functions will vary across ontogeny. Possibly, the scaling of these vital performances show different thermal sensitivities compared to MMR and RMR^78^. Here, MMR was measured using a standard chase protocol limiting our interpretation—possibly MMR induced following prolonged swimming or maximum feeding could lead to different temperature effects on *b*_MMR_, *b*_AAS_, and *b*_FAS_. Still, the consistent *b*_MMR_ ≈ *b*_RMR_ suggest that larger barred surfperch did not have reduced aerobic capacity during acute warming.

If the scaling slope for FAS was negative, this would indicate that larger surfperch experience a greater aerobic constraint than smaller ones^48^. However, we found a slightly positive scaling of FAS across acute temperatures. In another perch species (European perch), FAS scaled negatively (*b*_FAS_ = − 0.033) when acclimated to an optimal temperature (15 °C) for three weeks, and decreased further to *b*_FAS_ = − 0.067 at a suboptimal temperature (28 °C)^71^. In contrast, *b*_FAS_ ≈ 0.01 and did not change in leopard coral grouper that were acclimated to 28.5 °C and 33 °C for 3–5 days^79^. This discrepancy between studies might be due to acclimation versus the acute nature of temperature change or indicate species-specific responses to temperature change.

The aerobic metabolic capacity of fishes depends on the function of their heart, which plays a key role to ensure sufficient O_2_ supply to meet demand at the tissues. The heart is also often the first organ system that fails in fish under thermally challenging conditions^49,50,80^. Thus, we predicted that maximum heart rates would scale negatively with mass, and specifically that increasing temperature would have a negative effect on *f*_Hmax_ scaling slopes. Here, we found that larger individuals had lower *f*_Hmax_ than smaller ones, although the scaling relationship was consistent across temperatures and weak (*b* = − 0.05). In contrast, various meta-analysis and modelling studies have demonstrated that heart rates scale with more negative slopes in mammals at rest^39,40^ (*b*_*f*H_ = − 0.25) and during exercise^81^ (*b*_*f*H_ = − 0.187), birds at rest^39^ (*b*_*f*H_ = − 0.28) and during flight^81^ (*b*_*f*H_ = − 0.187), lizards^82^ at 30 °C (*b*_*f*H_ = − 0.15), and terrestrial snakes^37^ at 25 °C (*b*_*f*H_ = − 0.229). A recent experimental study on five cetaceans species reported heart rate scaling slopes between *b*_*f*H_ = − 0.16 and − 0.34^36^. However, the mild scaling of *f*_Hmax_ in our study aligns with previous findings in fishes^34,41,83^. A study using the same experimental protocol reported *b*_*f*Hmax_ = − 0.1 in redband trout^83^. Additionally, a statistically significant body size effect on *f*_Hmax_ was found in adult Arctic char^84^, adult brown trout^84^, Baltic herring embryos^9^, and on the field *f*_H_ in adult Chinook salmon^85^. A study on Atlantic salmon parr (~ 11 g) and post-smolts (~ 300 g) at 20 °C measured *f*_Hmax_ of 157 beats min^−1^ and 130 beats min^−1^, respectively^86^. Assuming these mean values comply with the *f*_Hmax_ mass scaling relationship, it would result in *b*_*f*Hmax_ ~ − 0.06, a very similar slope to ours (*b*_*f*Hmax_ = − 0.05). In comparison to Atlantic salmon, we measured lower *f*_Hmax_ at 20 °C, specifically ~ 123 beats min^−1^ in fish < 15 g and ~ 96 beats min^−1^ in > 200 g fish, which may be attributed to the lower athleticism of perch^22^. However, our PEAK_*f*Hmax_ values for ~ 200 g fish (~ 122 beats min^−1^) compare well to 115 beats min^−1^ in ~ 450 g European perch^56^ (note the difference in methods). It is worth noting that in our study PEAK_*f*Hmax_ did not significantly relate to body mass, but it could be explained by the fact that PEAK_*f*Hmax_ was achieved at different temperatures across individuals. The biological significance of the negative scaling of *f*_H_ and its temperature dependence is not clear yet, but certainly important to consider within and across species^14^.

The temperature at which the steady increase of *f*_Hmax_ with acute warming begins to slow (T_AB_), the *f*_Hmax_ peaks (T_PEAK_), and the heartbeat becomes arrhythmic (T_ARR_) all indicate functional thermal limits in fish^16^. Surprisingly, larger barred surfperch (~ 200 to 250 g) had higher cardiac thermal tolerance than young-of-the-year (~ 10 to 15 g). Similarly, a life-stage specific difference in T_AB_ and T_PEAK_ has been found in Atlantic salmon^86^. Specifically, the parr (~ 11 g) had lower T_AB_ by ~ 2.5 to 5 °C and higher T_PEAK_ by ~ 2.5 to 3 °C, but no differences in T_ARR_ compared to post-smolts (~ 300 g)^86^. In our study, T_AB_, T_PEAK_, and T_ARR_ were all lower in juveniles (< 50 g)^77^ compared to adults by an average of 1.62 °C, 1.79 °C, and 1.72 °C, respectively. Both studies suggest that intra-specifically, the optimal temperatures for cardiac performance are lower in smaller fish. In contrast, a study on cardiorespiratory capacity in adult Chinook salmon tested within 13 to 25 °C found cardiac arrhythmias at 25 °C only in the largest individuals (2.1–5.4 kg size range)^62^, and thus their results would suggest that the largest adults had lower thermal tolerance. The discrepancy between studies could be due to species- and life-stage-specific physiologies or methods used to measure *f*_H_. Still, the empirical evidence suggests that acute TPCs of *f*_Hmax_ is a plastic trait^54,55,87–89^ that varies across species^14^, life stage^86^, and body size.

While outside of the scope of this study, other physiological and morphological factors could help identify the aerobic limitations associated with body mass and temperature. In fish under warming, increasing heart rates is the dominant mechanism to achieve higher cardiac output, CO^14^. However, *f*_H_ is not the only performance contributing to CO (CO = *f*_H_ * *V*_S_; mL min^−1^; *V*_S_ = cardiac stroke volume). Aerobic metabolism and cardiac output are directly linked by Fick’s equation, which states that oxygen uptake rate (i.e., MR) is a product of *V*_S_, *f*_H_, and the difference between the O_2_ content in arterial and venous blood: MR = *f*_H_ * *V*s * (C_a_O_2_—C_v_O_2_); MR = CO * (C_a_O_2_—C_v_O_2_), where C_a_O_2_ and C_v_O_2_ are oxygen content (ml dl^−1^) in arterial and venous blood, respectively^14^. Scaling of oxygen content in blood, CO and *V*_S_ are not well established in fishes^62^. However, White and Kearney (2014) outlined that the discrepancy between the scaling of MR and *f*_H_ can be explained by the scaling of *V*_S_ because increased MR can be achieved by increasing either (or both) *f*_H_ and *V*_S_, e.g., assuming reciprocal relationships *b*_MR_ = 0.75 and *b*_*f*H_ = − 0.25, then *b*_*V*s_ ≈ 1 [*b*_MR_ (0.75) = *b*_*V*s_ (1) + *bf*_Hmax_ (− 0.25)]^20,90^. Thus, our estimated *b*_MR_ = 0.81 and *b*_*f*H_ = − 0.05 would suggest a scaling slope of ~ 0.86 for *V*_S_ [*b*_MR_ (0.81) = *b*_*V*s_ (0.86) + *bf*_Hmax_ (− 0.05)]. Further, in fishes, the cardiac stroke volume is positively correlated with relative ventricular mass^91^, which in our species scaled with *b*_VM_ = 0.85. Therefore, it would not be entirely surprising that *b*_*V*s_ ≈ 0.86 matching *b*_VM_ = 0.85. Larger hearts also allow for greater cardiac power output supporting higher MMR^92,93^, and so the strong hypoallometric scaling heart mass could partly explain why *b*_MMR_ ≠ 1, and *b*_MMR_ ≈ *b*_RMR_ in barred surfperch_._ For comparison, the ventricular mass scaled with* b*_VM_ = 0.939 in freshwater perches^94^, *b*_VM_ ~ 1 in salmonids^95^, and *b*_VM_ ~ 0.9 to 0.95 broadly across endotherms and ectotherms (mammals, birds, fishes, amphibians, and reptiles)^81,96^. These near isometric scaling slopes of VM across taxa could mechanistically support the trend that *b*_MMR_ ~ 1 > *b*_RMR_. Lastly, to our knowledge, only one study^97^ has estimated (and reported) mass effects on CO in fish species (rainbow trout) and found isometric scaling (*b*_*Q*_ = 1), while in mammals, CO can scale hypoallometrically and depend on activity (*b* < 0.90)^81^. It is critical to note that these cautiously outlined ideas are untested. Further, the contributions of *f*_H_ and *V*_S_ to CO and the scaling CO, *V*_S_, C_a_O_2_, C_v_O_2_ and their change with changing temperature in barred surfperch are unknown and warrant further study.

The concept of temperature-sensitive metabolic scaling has been incorporated in several hypotheses, which mainly focus on acclimation, maternal, and evolutionary effects of temperature on metabolic scaling^11,65,98,99^. It has been hypothesized that larger water-breathing ectotherms are more aerobically constrained under O_2_ demanding conditions, like warming and exercise, because of limited O_2_ supply^11,99^. An interspecific meta-analysis by Rubalcaba et al. (2020) developed a framework of scaling models that included biological and environmental factors that limit O_2_ supply (e.g., scaling of gill surface area, O_2_ partial pressures driving diffusion rates, ventilation rates), and showed that increasing activity (MMR) and increasing temperatures (species habitat temperature) interactively lead to decreased metabolic scaling slopes. Alternatively, the Gill Oxygen Limitation hypothesis predicts that relative gill surface area decreases with increasing body size, thus limiting sufficient O_2_ supply as fish grow larger^99^. In addition to gill surface area, other physiological mechanisms (e.g., ventilation frequency) have been suggested to interactively limit oxygen supply in larger fish, thus directly affecting scaling of resting metabolic rates^69,100^. Under warming, limitations in O_2_ supply are thought to disproportionally negatively impact larger fish, especially their MMR. At an intraspecific level, the *b*_MMR_ has been shown to increase^61^ or decrease^70,71^ following a non-monotonic pattern with increasing acclimation temperatures, thus providing mixed evidence. Oxygen supply mechanisms were not tested in our study and thus their contributions driving metabolic scaling relationships are unknown. Any discrepancies comparing inter-specific frameworks and intraspecific studies, including ours, may be due to the species examined, type of temperature exposure, and experimental approaches (e.g., MMR measurement methods). Even still, in our study, oxygen supply was unlikely to be a limiting factor irrespective of individual size and acute temperature exposure because *b*_MMR_ and *b*_RMR_ were consistent, the *b*_AAS_ > *b*_MMR_ ≈ *b*_RMR_ and *b*_FAS_ > 0 across acute temperatures. Studies examining the scaling of other pieces in the oxygen cascade^101^, will be useful for better understanding warming-associated constraints in fish across sizes.

Independent of fish’s body size, aerobic performance was maintained across an acute 10 °C range (12 to 22 °C) and had low thermal sensitivity (Q_10_ < 2). This is characteristic of eurythermal species like opaleye^45^, which share a similar coastal range to our study species, and other perch species (European perch^71,89^, yellow perch^72^). Notably, in barred surfperch, the RMR and MMR were somewhat low (mean 3.93 and 7.06 mgO_2_ min^−1^ 65 g^−1^ at 20 °C in wild-caught fish, respectively), leading to a low AAS (mean 2.90 mgO_2_ min^−1^ 65 g^−1^ at 20 °C), and FAS (≤ 2.27). These values are similar to those observed in a close relative, the striped surfperch (*Embiotoca lateralis*), where metabolic rate was ~ 1.3 mgO_2_ min^−1^ kg^−1^ swimming at 0.5 body lengths s^−1^ and ~ 3 mgO_2_ min^−1^ kg^−1^ swimming maximally in 11 °C^102^. Additionally, European perch had a similar AAS at 20 °C (~ < 3 mgO_2_ min^−1^ kg^−1^)^71,89^. Perch species may be generally classified as non-athletic due to their lifestyle and ecology, thus possessing a lower aerobic capacity^22^.

Aerobic and cardiac thermal performance of barred surfperch showed signs of decline above ~ 20 °C. Even though the barred surfperch’s AAS was maintained across the 10 °C range, their MMR did not continue to increase significantly from 20 °C to 22 °C, T_AB_ was ~ 20 °C, FAS was < 2 at 22 °C, T_PEAK_ was ~ 24 °C, and their hearts became arrhythmic at ~ 26 °C. Furthermore, we unexpectedly observed 50% mortality in lab-born juveniles under an acute 24 °C exposure (during a discontinued respirometry trial). Our results agree with those studies where T_AB_ and optimal temperature for aerobic metabolism overlap in fishes^12,49,103^. Altogether, the measured cardiac and metabolic performances indicate that aerobic capacity declined > 20 °C, the functional thermal limit was ~ 22 °C, and the acute upper thermal limits were likely between ~ 24 and ~ 26 °C in barred surfperch. South of our study location, in Baja California, the temperature in the surf zone can reach 24 °C lasting up to 16 h^104^. Thus, if the observed 50% mortality of juveniles at 24 °C is representative of barred surfperch, a northward shift in this species could be possible as their current suitable habitat from Northern California, USA, to Baja California, Mexico, constricts with coastal warming and temperature extremes^105^. Alternatively, perch from northern or southern habitats, or lab-born versus wild fish, may possess different functional and absolute thermal limits. Though, the population genetic structure, and developmental plasticity of barred surfperch are unknown.

Barred surfperch, a viviparous species, is a great study model for body size studies because fish of any life stage occupy the same thermally dynamic surf zone ecosystems with distinct seasonal, diurnal (acute, hourly), and anomalous (heatwaves and upwelling) temperature changes (Fig. 2). For instance, the juveniles that have lower acute cardiac thermal tolerance may be particularly vulnerable under acutely increasing temperature (≥ 20 °C) in the wild. Additionally, the geographic range of barred surfperch intersects 52 Marine Protected Areas^106^ with common temperature conditions from ~ 12 °C to ~ 23 °C^77,107^ (Fig. 2), their economic and recreational value is continuously increasing, and they fulfill key ecological roles by connecting aquatic and terrestrial fauna and flora communities. Therefore, this study system also provides the opportunity to integrate ecophysiology into management and conservation.

This study specifically explored the effects of ecologically relevant acute temperatures as opposed to the effects of a multi-week thermal acclimation^44,89^. Although barred perch must respond to both acute (hourly) and seasonal temperature changes, acute timescales (2 °C h^−1^) are particularly relevant for coastal California fishes. They experience high daily thermal fluctuations during the summer and early fall and are exposed to rapid temperature changes associated with coastal upwelling in the spring and fall^108^. When encountering acute temperature change, fish predominantly modulate their heart rate to ensure adequate oxygen delivery to the tissues with changing metabolic needs, and thus *f*_Hmax_ is a highly relevant target mechanism in studying thermal physiology in fish^19^. Further, in fishes, *f*_Hmax_ can acclimate rapidly, within the first 48 to 72 h after temperature change^55^, while metabolic rates can take from 72 h to ~ 5 days to stabilize^109,110^. Our results suggest that barred surfperch must be able to physiologically respond to acute temperature change within hours in the wild, are physiologically able to maintain their AAS across a 10 °C range up to ~ 22 °C. Possibly, the phenotypic changes following full muti-week physiological thermal acclimation^44,53,89^ could underscore different scaling relationships compared to acutely exposed fish. Further, this study suggests that thermal history and developmental plasticity could play an important role in aerobic capacity and thermal tolerance. Specifically, lab-born juveniles had lower cardiac thermal tolerance, and thus aerobic capacity compared to wild-caught juveniles which may have been due to different thermal history during development. Lab born-juveniles were acclimated to static 16 °C throughout their development in the lab which could have reduced their physiological capacity and plasticity in response to acute temperature change, unlike their wild counterparts that experience a wide breath of acute and seasonal temperatures (Fig. 2).The next step may be to explore the scaling of time to acclimation and acclimation capacity of metabolism, cardiac function, and other physiological performances across organ systems and species^52,53^.

In conclusion, some studies with ectotherms have found that larger individuals are more vulnerable to warming than smaller ones^2,11,79^. However, this study did not find any warming-associated constraints in large fishes. Consistent mass scaling of metabolic performance (*b*_RMR_ = *b*_MMR_ 0.81; O_2_ demand) together with negative and weak scaling of maximum heart rates (*b*_*f*Hamx_ = − 0.05; O_2_ supply mechanism) suggested that inadequate oxygen supply is an unlikely constraint on cardiac performance and metabolic rates in barred surfperch under warming. In fact, larger barred surfperch had superior cardiac thermal tolerance compared to smaller counterparts, as indicated by positive scaling of T_AB_, T_PEAK,_ and T_ARR_ (*b* ~ 0.03). Barred surfperch currently experience temperatures close to their acute functional thermal limit. Together, this study suggests that body size vulnerability to warming is nuanced and not a universal trait.

## Materials and methods

All data and statistical analyses were done in R v. 4.2.0 (2022). All animal handling and holding procedures were compliant with Protocol # 945 approved by the University of California, Santa Barbara Institutional Animal Care and Use Committee, and fish were collected under approved California Department of Fish and Wildlife collection permits. All methods were performed in accordance with the relevant guidelines and regulations. The study is reported in accordance with ARRIVE guidelines.

### Animals

Barred surfperch, *Amphistichus argenteus* (N = 61; ~ 5 to 700 g) were caught in the beach zone in Santa Barbara County using a seine net (50 ft with catch bag, 30 ft no catch bag) or hook and line in April through May in 2021 (Spring experiments), and July 2021 (Summer experiments). Fish were transported to the University of California, Santa Barbara, in aerated filtered ambient flow-through seawater (> 80% air saturation). Wild-caught fish were kept in various size tanks (409 L, 303 L, and 94.6 L tanks; 2–13 fish per tank). Fish were grouped by size to avoid social stress between differently sized individuals. Barred surfperch are livebearers giving birth to fully developed juveniles in spring and early summer^77^. Five females were collected gravid during spring experiments (confirmed during dissections), giving birth to 79 juveniles (~ 2–3 g) in the laboratory (parent females to each offspring could not be assured; > 1 gravid female per tank). Laboratory-born juveniles were transferred to 37.9 L tanks at 16 °C (N = 6 to 12 fish per tank). Fish were kept at 16 °C (± 1.0 °C) using mixed chilled or heated filtered ambient seawater at > 90% air saturation under a 10D:14L light cycle. Water quality was tested weekly using commercial test kits (NO_2_^−^ < 0.25 ppm, NO_3_^−^ < 20 ppm_,_ NH_3_ < 0.25 ppm, pH = 7.7 to 8.0, all matching ambient ocean seawater). Fish were fed daily to satiation with a diverse carnivorous diet (fresh or thawed mussels, thawed shrimp, squid, scallops, frozen brine shrimp, fresh sand crabs, *Emerita analoga*). Feeding was discontinued ~ 36 h before the respirometry trial. Fish were tagged with a visible fluorescent Elastomer tag (Northwest Marine Technology, Inc) and provided at least a 3-day recovery between trials.

### Aquatic intermittent-flow respirometry

Methods reported following published guidelines for intermittent respirometry^111^. Oxygen consumption rates (MO_2_), a proxy for metabolic rates, were measured using intermittent flow respirometry across four acute temperatures in a repeated measurement design. Fish were first tested at 16 °C (acclimation temperature) and then after an acute temperature change (2 °C h^−1^) at 20 °C, 12 °C, and 22 °C (one round of trials was done in shuffled order, confirming it did not affect results). The ramp rate was selected to mimic ecologically relevant acute thermal events in kelp forests and nearshore environments along the Pacific coastline where barred surfperch live (Fig. 2a,c). The temperature was changed directly in housing tanks by adjusting incoming flow rates of chilled (10 °C) and warm (~ 20 to 22 °C) filtered seawater and by using submersible heaters with control unit; the same approach was used to maintain temperatures at their target level during respirometry. Fish were kept at their treatment temperature for at least 30 min before chasing (± 1 °C). Because only two fish could be chased at the time, the time that fish spent in acutely changed temperature before the chase varied between 30 to approx. 90 min. A higher, 24 °C acute temperature treatment was initially considered but led to 50% mortality (n = 4/8) in a group of laboratory-born juveniles. This treatment was discontinued. After an overnight respirometry trial, fish were returned to their housing tanks, and the temperatures were brought back to 16 °C at the same rates (i.e., 2 °C h^−1^).

Respirometry setup consisted of custom-built plastic chambers of various sizes (minimum 0.272 L, maximum 32.120 L), allowing for 19.2 to 93.9 net respirometer volume to fish body mass ratio^112^. Each chamber had one recirculating water loop and one flush loop, both connected to flow-controlled pumps (Ehaim compactON, Eheim universal; EHEIM GmbH & Co. KG. Deizisau, Germany). A robust fiberoptic oxygen sensor (PyroScience GmbH, Aachen, Germany) was placed in the recirculating loop. The temperature was controlled using submersible heaters and monitored using a Pt100 temperature probe (PyroScience GmbH, Aachen, Germany). Oxygen sensors and a temperature probe were connected to FireSting Optical Oxygen Meter (PyroScience GmbH, Aachen, Germany). All respirometry trials were performed in an environmental chamber, minimizing disturbance during the trial.

MMR was elicited following a standard chase and air exposure protocol (3-min chase, 1-min air exposure)^73^. Chase tanks were selectively sized to allow bursting in all fish. Immediately after air exposure, fish were placed in the respirometry chamber, and their metabolic rates were recorded (MMR_CHASE_). Fish recovered in respirometers overnight on an automated 15-min cycle of flush: measure (11:4, 10:5, 9:6, or 8:7 min, according to fish mass to chamber volumes ratio and temperature), yielding > 60 MO_2_ measurements. During trials, oxygen levels were at > 70% air saturation and within ± 1 °C of the experimental temperature. After the respirometry trial, fish were weighed to the nearest 0.01 g (fish < 60 g) or nearest 0.1 g (fish > 60 g), measured for length (cm), sexed when possible, and returned to their housing tanks. Chases were performed between 0900 and 1300 h, and fish were removed from the chambers between 0700 and 0900 h. Background respiration by microorganisms was measured in empty respirometry chambers before and after each trial. The background respiration levels were mean of 10% (median = 6.6%) of individuals respiration.

### Arrhenius breakpoint temperature

Arrhenius breakpoint temperature (ABT) tests were set up and carried out following established methods^12^ previously used on marine fish^45,87^. We used a custom-built ABT test tank (33L × 20.5W x 22H cm, Igloo Playmate Elite Cooler 16 qt, filled to 12 L) that contained *i*) an elevated sling with silicone fish beds (n = 1 to 2; each with plastic straps to secure fish), *ii*) a circulation loop with flow control valve and soft plastic tubing to irrigate the gills of fish during the trial, *iii*) two air stones to keep oxygen levels at > 90% air saturation, and *iv*) heating coil connected to a Polystat recirculating heater/chiller unit (Cole-Palmer, Vernon Hills, IL, USA) to regulate the water temperature. The test tank was filled with seawater with a maintenance dose of anesthetic (65 mg MS-222 1 g L^−1^ buffered with NaHCO_3_^−^ at 1:1 or higher ratio). Flow rates across the gills were kept between 25 ml s^−1^ and 55 ml s^−1^, depending on fish size.

Individual fish selected for the ABT test were anesthetized in 80 mg MS-222 1 g L^−1^ buffered with NaHCO_3_^−^, weighed to the nearest 0.01 g, and securely placed on the fish bed (laying on the side slightly tilted down and flow passing the gills). A stainless-steel Needle Tip Electrode (ADInstruments INC, Colorado Springs, CO, USA) was placed just under the skin on the ventral surface by the pericardium to detect an ECG signal. The ECG signal was amplified and filtered using Dual Bio Amp and Powerlab data acquisition system (ADInstruments INC, Colorado Springs, CO, USA) at the following settings: 60 Hz Notch filter; Mains filter; Low-Pass: 2Kz; High Pass: 10 Hz; Range: 2 mV. No more than four individuals were tested at the time.

Once all fish were positioned in the test tank, they were left undisturbed for a 30-min equilibration period at 16  °C. Atropine sulfate (1.2 mg kg^−1^ in 0.9% NaCl) was then injected intraperitoneally to block vagal tone, which was followed by a 15-min equilibration period. Then, isoproterenol (4 μg kg^−1^ in 0.9% NaCl) was injected intraperitoneally to maximally stimulate *β*-adrenoreceptors, which was followed by the final 15-min equilibration period. The temperature was then increased by 1 °C every 6 min (ramp rate 1 °C 5 min^−1^) and held steady for 1 min. Temperature and maximum heart rate (*f*_Hmax_) for data analysis were recorded within the last 30 s of each 1 °C increment (i.e., minutes 5:30 to 6:00 of each temperature ramp interval). Incremental temperature ramp was continued until the heart became arrhythmic (T_ARR_), defined by a clear transition from rhythmic to arrhythmic beating, or until missed QRS peak underlying a precipitous decrease in heart rate^12^. This was an endpoint of the ABT test, and the fish was immediately removed from the anesthetic, euthanized, and their ventricle was excised and weighed (nearest 0.001 g). All fish were also weighed to the nearest 0.01 g (fish < 60 g) or nearest 0.1 g (fish > 60 g), measured for length (cm), and sexed when possible.

## Data analysis

Aerobic metabolism measurements were analyzed, and metabolic capacity metrics were estimated using custom-written functions in R (https://github.com/kraskura/AnalyzeResp_0). The decreasing dissolved O_2_ content (mgO_2_ L^−1^) collected during respirometry trials during each measurement cycle was plotted over time (min) and fitted with simple linear regression (‘lm’ in R). All linear regressions were visually assessed for quality and linearity. Only regressions with R^2^ > 0.96 were used for analysis. The selected regression slopes were used to calculate individuals’ oxygen uptake (MO_2_, mgO_2_ min^−1^), a proxy for metabolic rate following: MO_2_ = [(*m*_fish_*V) − (*m*_background_*V)], where slope (m) is the decline of O_2_ content (mgO_2_ L^−1^) over time (min), and V is the volume of the respirometer (L).

Maximum metabolic rate (MMR) is often elicited after a strenuous swim, chase, or during digestion^74–76^, resting, or minimum metabolic rate is measured in post-absorptive, non-reproductively active, resting individuals^113^. Barred surfperch behaviorally respond to various types of exercise by laying down on their side (personal observation in field and laboratory). This was observed in the respirometers immediately after the chase, likely contributing to why 74% of MMR values were observed during spontaneous overnight activity (lights turning on or off^71^); still, the values were comparable with those after the chase (Supplementary Fig. S5 online). Similarly, studies on a similar fish species, European perch, noted that MMR was observed via turning on a light-source and gently tapping the respirometer^71,114^. Additionally, five adult females from spring experiments were reproductively active. Males are reproductively active in the fall, but mature gonads were not observed during dissections. Acknowledging these constraints, the AAS was calculated using RMR as a baseline of AAS, and MMR was the highest MO_2_ recorded across ≥ 3 min at any time point during the trial. RMR was calculated as the mean of the 10 lowest estimated MO_2_ values after excluding the five lowest values from the entire trial^113^. We excluded the first 60 s of each measurement (mixing or wait period) but ensured that all MO_2_ measurements were at least 180 s long. The factorial aerobic scope (FAS) was calculated as FAS = MMR / RMR.

The electrocardiogram data from Arrhenius breakpoint temperature trials were analyzed directly in LabChart 8 (ADInstruments INC, Colorado Springs, CO, USA). Maximum heart rate (ƒ_Hmax_) was calculated for each 1 °C increment during 15 s (± 2 s) visually assessed measurements. The heart rate (beats min^−1^) was calculated by automated ECG analysis tools available in LabChart 8, and each fit was confirmed visually. The ƒ_Hmax_ values recorded before T_ARR_ were used to establish acute TPC of maximum heart rate for each fish, which was then used to calculate several cardiac performance metrics. The breakpoint at which the incremental increase in individual fish *f*_Hmax_ changed rates was estimated on regression *ln*(ƒ_Hmax_) ~ 1000/Temperature (in Kelvin) using the segmented function in R (package ‘segmented’^115^) with parametric bootstrap (n = 100 boot samples). The breakpoint estimate was not included when: (i) the confidence interval of the estimated breakpoint exceeded ± 1.5 °C (n = 2), (ii) the breakpoint was not statistically identified (n = 1). The temperature ( °C) corresponding to the breakpoint was calculated and is referred to as T_AB_. The PEAK_*f*Hmax_ refers to the highest ƒ_Hmax_ recorded across all temperatures (i.e., the peak of the acute TPC of maximum heart rate), and the temperature at PEAK_*f*Hmax_ is referred to as T_PEAK_.

Morphometrics, sample sizes, and sex, when available, are provided in Supplementary Table S5 online. Barred surfperch are sexually dimorphic, but the sex-specific characteristics are not fully developed until fish reach approximately > 7 g in size.

### Statistical analysis

Mass scaling relationships were estimated for metabolic performance metrics (RMR, MMR, AAS, FAS), for cardiac physiology performances (ƒ_Hmax,_ T_AB,_ T_ARR,_ T_PEAK,_ and PEAK_*f*Hmax_ ), and between ventricle mass and body mass. All performance metrics and body mass (BM, kg) were natural *log*-transformed to comply with the linear homoscedastic form of the scaling law (Fig. 1; *ln*(performance) = *ln*(*a*) + b*ln(BM)) where *b* = scaling exponent defining scaling slope, and *ln*(*a*) = scaling coefficient, or the intercept.

Consistency of scaling relationships across temperature treatments for RMR, MMR, AAS, FAS, and ƒ_Hmax_ were determined using mixed-effect linear models (‘lmer’ in ‘lme4’ package^116^). We included a random intercept effect of individual fish to account for repeated measures. The independent explanatory variables were body mass (*ln*BM; continuous), temperature (°C; categorical), origin (laboratory-born, wild-collected fish; categorical), and sex (when available; categorical). Including temperature as a categorical variable (fixed effect) allowed us to test for differences in scaling relationships (slope and intercept) across temperature treatments, while still detecting the shape of thermal performance curves (n = 4 temperatures are insufficient to robustly estimate TPC on continuous scales). We considered size class (< 50 g “juvenile”, > 50 g “adult”) as an explanatory variable. Since it had no significant effect, it was not further considered (data-deficient sets). Complementary models were compared using BIC, where the model with the lowest BIC score was accepted as the best fit^24^ (Supplementary Table S1 online). Cardiac physiology measures (T_AB,_ T_ARR,_ T_PEAK,_ and PEAK_*f*Hmax_) and ventricle mass (VM) were independent and modeled using simple, generalized linear models (‘lm’, ‘glm’). Supplementary to these analyses, we estimated temperature-specific (12, 16, 20, and 22 °C) scaling relationships for RMR, MMR, AAS, FAS, and *f*_Hmax_; refer to supplementary material online for models and results. All model residuals were normally distributed, and fits were visually assessed.

The significance of body mass, temperature, sex, or origin was tested using Type II ANOVA (‘car’ package^117^). The significance of performance between different temperatures was tested using the Tukey post hoc test with the Kenward-Roger degrees of freedom method (‘emmeans’^118^). To evaluate only temperature effects on metabolic performances (RMR, MMR_,_ AAS, and FAS) and *f*_Hmax_, we mass-normalized individual values to represent performance of a mean size fish, 65 g (i.e., removed hypoallometric scaling effects^90^) using scaling relationships from best mixed effect models. Metabolic performances are expressed in mass-specific units (mgO_2_ min^−1^ 65 g^−1^). The mass-independent mean values were used to calculate the temperature sensitivity coefficient, Q_10,_ following Q_10_ = R2 / R1^(10/ (T2—T1)^, where R1 and R2 are the average performance values at their corresponding temperatures, T1 and T2. Lastly, we used a 95% confidence interval (CI) and standard error (SE) to report the error of mean estimates. All reported values are maximum likelihood estimates. The significance was accepted at P < 0.05.

## Data and code availability

The original data files, and all analysis and statistics scripts are available on GitHub: https://github.com/kraskura/KK_etal_perch_scaling_temp. The respirometry data were analyzed using custom functions documented on Github: https://github.com/kraskura/AnalyzeResp_0.

### Supplementary Information

Supplementary Information.
