# Supplementary material for: Body size and temperature affect metabolic and cardiac thermal tolerance in fish
Source: Sci Rep. 2023 Oct 19;13:17900. doi: 10.1038/s41598-023-44574-w (PMC10587238; doi:10.1038/s41598-023-44574-w)
Supplement: Supplementary file 1 — Supplementary Information. [file 41598_2023_44574_MOESM1_ESM.pdf]

Supplementary material to:

"Body size and temperature affect metabolic and cardiac thermal tolerance in fish."
Krista Kraskura, Emily A. Hardison, and Erika J. Eliason

Table S1. Linear models for metabolic scaling and results of model selection (BIC). 2
Table S2. Estimated mass scaling relationships of all measured physiological performances. 4
Table S3. ANOVA and Post-hoc results for best fit models. 5
Table S4. Mass-independent mixed model estimates of physiological performances. 6
Table S5. Summary of physiological tests complete. 7
Figure S1. Temperature-specific hypometric scaling of maximum and resting metabolic rates across acute temperatures in barred surfperch. 8
Figure S2. Negative temperature-specific scaling of maximum heart rate (f_{Hmax}) in barred surfperch across acute temperatures 9
Figure S3. Mass scaling of cardiac thermal performance indices. 10
Figure S4. Scaling of ventricular mass. 11
Figure S5. Comparison of individual maximum metabolic rate measurements. 12

Temperature specific scaling relationships

Methods: Temperature treatment specific (12, 16, 20, and 22ºC) scaling relationships for RMR, MMR, AAS, and FAS, and f_{Hmax} were established using simple linear regressions ('lm', 'glm' in R). In these models, the independent explanatory variables were body mass (*ln*BM), temperature (ºC), origin (laboratory-born, wild-collected fish) and sex (when available).
Value and outcomes: Estimated scaling relationships for each temperature subgroup were $b_{MMR} > b_{RMR}$ and $b_{AAS} \sim 0.88$ for all acute temperature treatments.

Tables

Table S1. Linear models for metabolic scaling and results of model selection (BIC).

BM = body mass, Orig = origin (lab born or wild collected), T = temperature ºC, ID = unique fish ID, repeat measures, categ = categorical variable, k = the number of parameters estimated using maximum likelihood. LM = simple linear model, no repeated measures.

Model	Hypo-Theses	k	BIC	ΔBIC
Maximum metabolic rates (MMR):				
log(MMR) ~ log(BM) + T + Orig + (1|ID)	Temperature treatment (T, categ.), repeat measures (1|ID), Sex (categ.) and Orig (categ.)	8	-195.9410	3.25899
log(MMR) ~ log(BM) + T + Sex + Orig + (1|ID)		9	-175.2323	23.96777
log(MMR) ~ log(BM) + T + Sex + (1|ID)		8	-178.3186	20.88146
**log(MMR) ~ log(BM) + T + (1|ID)**		**7**	**-199.2000**	**0**
log(MMR) ~ log(BM) * T + (1|ID)		10	-183.4366	15.76344
log(MMR) ~ log(BM)	temperature specific LM. 12, 16, 20, 22ºC, no covariates			
Minimum metabolic rates (RMR)				
**log(RMR) ~ log(BM) + T + Orig + (1|ID)**	T (ºC, categ.), repeat measures (1|ID), Sex (categ.), Orig (categ.)	**8**	**-41.75825**	**0**
log(RMR) ~ log(BM) + T + Sex + Orig + (1|ID)		9	-29.11491	12.64334
log(RMR) ~ log(BM) + T + Sex + (1|ID)		8	-25.27646	16.48179
log(RMR) ~ log(BM) + T + (1|ID)		7	-36.90996	4.84829
log(RMR) ~ log(BM) * T + (1|ID)		10	-21.87646	19.8818
log(RMR) ~ log(BM)	temperature specific LM. 12, 16, 20, 22ºC, no covariates			
Absolute Aerobic scopes (AAS = MMR - RMR)				
log(AAS) ~ log(BM) + T + Orig + (1|ID)	T (ºC, categ.), repeat measures (1|ID), Sex (categ.), Orig (categ.)	8	146.7398	3.46968
log(AAS) ~ log(BM) + T + Sex + Orig + (1|ID)		9	151.5921	8.32202
log(AAS) ~ log(BM) + T + Sex + (1|ID)		8	147.5571	4.28704
**log(AAS) ~ log(BM) + T + (1|ID)**		**7**	**143.2701**	**0**
log(AAS) ~ log(BM) * T + (1|ID)		10	158.8107	15.54062
log(AAS) ~ log(BM)	temperature specific LM. 12, 16, 20, 22ºC, no covariates			
Factorial Aerobic scope (FAS = MMR / RMR)				
**log(FAS) ~ log(BM) + T + Orig + (1|ID)**	T (ºC, categ.), repeat measures (1|ID), Sex (categ.), Orig (categ.)	**8**	**-132.0578**	**0**
log(FAS) ~ log(BM) + T + Sex + Orig + (1|ID)		9	-115.8804	16.17744
log(FAS) ~ log(BM) + T + Sex + (1|ID)		8	-114.9501	17.10772
log(FAS) ~ log(BM) + T + (1|ID)		7	-129.8056	2.25222
log(FAS) ~ log(BM) * T + (1|ID)		10	-115.1419	16.91589
log(FAS) ~ log(BM)	temperature specific LM. 12, 16, 20, 22ºC, no covariates			
Maximum heart rate (beats min^{-1}) across temperatures				

**log(f_{Hmax}) ~ log(BM) + T + (1|ID)**	T (ºC, categ.), repeat measures (1|ID), no covariates	**16**	**-733.2281**	**0**
log(f_{Hmax}) ~ log(BM) * T + (1|ID)		28	-687.8539	45.37416
log(f_{Hmax}) ~ log(BM) + (1|ID)		4	-224.0942	509.13385
Temperature at which the heart became arrhythmic				
log(T_{ARR}) ~ 1	*NULL*	2	-67.36079	4.2596
**log(T_{ARR}) ~ log(BM)**	T (ºC, categ.), repeat measures (1|ID), Sex (categ.), Orig (categ.)	**3**	**-71.62039**	**0**
log(T_{ARR}) ~ log(BM) + Orig		4	-68.94095	2.67944
log(T_{ARR}) ~ log(BM) + Sex		4	-54.08455	17.53584
log(T_{ARR}) ~ log(BM) + Sex + Orig		5	-53.68086	17.93953
Temperature at which fish had the peak maximum heart rates				
log(T_{PEAK}) ~ 1	*NULL*	2	-61.76246	4.43588
**log(T_{PEAK}) ~ log(BM)**	T (ºC, categ.), repeat measures (1|ID), Sex (categ.), Orig (categ.)	**3**	**-66.19834**	**0**
log(T_{PEAK}) ~ log(BM) + Orig		4	-62.91077	3.28757
log(T_{PEAK}) ~ log(BM) + Sex		4	-50.22768	15.97066
log(T_{PEAK}) ~ log(BM) + Sex + Orig		5	-47.86668	18.33166
Breakpoint temperature of maximum heart rates				
log(T_{AB}) ~ 1	*NULL*	**2**	-61.80491	11.56258
log(T_{AB}) ~ log(BM)	T (ºC, categ.), repeat measures (1|ID), Sex (categ.), Orig (categ.)	3	-71.29383	2.07366
**log(T_{AB}) ~ log(BM) + Orig**		**4**	**-73.36749**	**0**
log(T_{AB}) ~ log(BM) + Sex		4	-53.76421	19.60328
log(T_{AB}) ~ log(BM) + Sex + Orig		5	-54.47289	18.8946
The absolute peak maximum heart rates recorded at any temperature				
**log($PEAK_{fHmax}$) ~ 1**	*NULL*	**2**	**-49.52628**	**0**
log($PEAK_{fHmax}$) ~ log(BM)	T (ºC, categ.), repeat measures (1|ID), Sex (categ.), Orig (categ.)	3	-46.4506	3.07568
log($PEAK_{fHmax}$) ~ log(BM) + Orig		4	-44.09186	5.43442
log($PEAK_{fHmax}$) ~ log(BM) + Sex		4	-38.18762	11.33866
log($PEAK_{fHmax}$) ~ log(BM) + Sex + Orig		5	-39.11335	10.41293
Ventricular mass (VM, kg)				
log(VM)~log(BM)	VM = ventricular mass, no covariates			

Table S2. Estimated mass scaling relationships of all measured physiological performances. All temperatures are included as a categorical explanatory variable. SE = standard error of mean.

Physiological performance	n indiv. (n obs)	T, ºC	Scaling slope, *b* (SE) {$CI_{2.5\%}$, $CI_{97.5\%}$}	Intercept, *ln*(*a*) (SE)
MMR (mgO_2 min^{-1})	66	12	0.817 (0.017) {0.78, 0.85}	1.036 (0.064)
	76	16	0.809 (0.014) {0.78, 0.84}	1.231 (0.054)
	68	20	0.798 (0.012) {0.77, 0.82}	1.401 (0.043)
	28	22	0.843 (0.016) {0.81, 0.87}	1.641 (0.062)
RMR (mgO_2 min^{-1})	66	12	0.761 (0.024) {0.71, 0.79}	0.130 (0.087)
	74	16	0.750 (0.022) {0.71, 0.79}	0.454 (0.084)
	66	20	0.747 (0.015) {0.72, 0.78}	0.696 (0.053)
	27	22	0.813 (0.027) {0.76, 0.87}	1.089 (0.103)
Absolute aerobic scope (mgO_2 min^{-1}) (MMR – RMR)	66	12	0.878 (0.027) {0.83, 0.93}	0.534 (0.098)
	74	16	0.890 (0.029) {0.83, 0.95}	0.618 (0.108)
	66	20	0.883 (0.033) {0.82, 0.95}	0.754 (0.117)
	27	22	0.886 (0.038) {0.81, 0.96}	0.736 (0.144)
Factorial aerobic scope (MMR / RMR)	66	12	0.056 (0.018) {0.02, 0.09}	0.906 (0.065)
	74	16	0.060 (0.017) {0.03, 0.09}	0.780 (0.063)
	66	20	0.052 (0.015) {0.02, 0.08}	0.713 (0.055)
	27	22	0.028 (0.020) {-0.01, 0.07}	0.546 (0.076)
f_{Hmax} (beats min^{-1})	27	16	-0.068 (0.009) {-0.09, -0.05}	4.270 (0.032)
	30	20	-0.056 (0.010) {-0.08, -0.04}	4.575 (0.035)
	29	22	-0.045 (0.012) {-0.07, -0.02}	4.700 (0.043)
	26	24	-0.036 (0.012) {-0.06, -0.01}	4.780 (0.040)

Table S3. ANOVA and Post-hoc results for best fit models. Post hoc statistics are noted for models with significant temperature main effects. Origin = lab-born or field-collected fish. Null model was best predicting $PEAK_{fHmax}$, not independent predictor variables.

	***Ln*(BM)**		**Temperature ºC**		**Origin**	
Mixed model	*χ^2 (df)*	*P-value*	*χ^2 (df)*	*P-value*	*χ^2 (df)*	*P-val*
**MMR**	6989.51 (1)	< 0.0001	463.88 (3)	< 0.0001	--	--
Post-hoc results			12 vs 16ºC	***		
			12 vs 20ºC	***		
			12 vs 22ºC	***		
			16 vs 20ºC	***		
			16 vs 22ºC	***		
			20 vs 22ºC	0.180		
**RMR**	1568.9 (1)	< 0.0001	515.971 (3)	< 0.0001	11.035 (1)	0.0009
Post-hoc results			12 vs 16ºC	***		
			12 vs 20ºC	***		
			12 vs 22ºC	***		
			16 vs 20ºC	***		
			16 vs 22ºC	***		
			20 vs 22ºC	*0.018		
**AAS**	2268.224 (1)	< 0.0001	21.976 (3)	< 0.0001	--	--
Post-hoc results			12 vs 16ºC	0.707		
			12 vs 20ºC	**		
			12 vs 22ºC	*0.046		
			16 vs 20ºC	*		
			16 vs 22ºC	0.238		
			20 vs 22ºC	0.963		
**FAS**	1.567 (1)	0.2106	72.503 (3)	< 0.0001	8.013 (1)	0.0046
Post-hoc results			12 vs 16ºC	***		
			12 vs 20ºC	***		
			12 vs 22ºC	***		
			16 vs 20ºC	0.242		
			16 vs 22ºC	*		
			20 vs 22ºC	0.241		
**f_{Hmax}**	30.957 (1)	< 0.0001	1767.892 (12)	< 0.0001	--	--
Simple model	*F (df)*	*P-value*	*F (df)*	*P-value*	*F (df)*	*P-val*
**T_{ARR}**	8.122 (1)	0.0083	--	--	--	--
**T_{PEAK}**	8.359 (1)	0.0073	--	--	--	--
**T_{AB}**	8.851 (1)	0.0066	--	--	5.281 (1)	0.0306

Table S4. Mass-independent mixed model estimates of physiological performances. Summary of mixed model marginal mean performance estimates for fish adjusted to 65 g. These values were used to perform post-hoc analysis. *emm = estimated marginal mean, mass - specific. CV = coefficient of variation (SD / mean * 100 %).

Perfor-mance	Origin	T ºC	df	n	mean (emm*)	CV
**RMR**	Field-collected	12	94.1	44	2.133	17.80
		16	92.4	46	3.043	19.60
		20	91.9	48	3.928	13.10
		22	137.8	19	4.450	17.20
	Laboratory-born	12	81.7	22	2.581	16.50
		16	80.3	28	3.681	8.95
		20	81.8	18	4.751	9.77
		22	99.9	8	5.383	6.96
**MMR**	Field-collected	12	105.9	66	4.612	12.60
		16	104.8	76	5.774	12.20
		20	99.4	68	7.057	9.94
		22	158.0	28	7.502	7.77
**Absolute Aerobic scope**	Field-collected	12	108.9	66	2.362	22.80
		16	108.2	74	2.485	24.80
		20	103.1	66	2.898	24.80
		22	166.3	27	2.807	21.40
**Factorial Aerobic scope**	Field-collected	12	95.7	44	2.147	15.00
		16	93.8	46	1.879	15.80
		20	93.3	48	1.791	14.00
		22	143.8	19	1.676	13.10
	Laboratory-born	12	81.5	22	1.893	14.60
		16	79.8	28	1.656	11.60
		20	81.6	18	1.579	9.81
		22	101.8	8	1.477	5.75
f_{Hmax}	Field-collected	16	37.8	31	87.017	3.25
		17	38.4	34	92.399	3.42
		18	38.9	30	99.754	2.83
		19	38.9	30	106.713	2.54
		20	38.9	30	113.270	2.64
		21	38.9	30	119.165	3.25
		22	39.0	29	123.902	3.82
		23	39.6	25	127.768	3.53
		24	39.4	26	129.654	3.65

		25	40.1	23	132.057	5.57
		26	41.4	20	133.791	7.07
		27	66.2	7	122.883	23.90
		28	176.8	2	129.218	0.69

Table S5. Summary of physiological tests complete. N = sample size, F = female, M = male, *NA* = not available. Origin (laboratory-born or field caught).

Tests	Origin	Pregnancy	Size class	Sex	N	N (total)
Respirometry (MMR, RMR, AAS, FAS)	wild	No	Adult (> 50 g)	F	12	83
				M	21	
		No	Juvenile (< 50 g)	F	5	
				M	6	
				NA	1	
		Yes	Adult (> 50 g)	F	5	
	lab	No	Juvenile (< 50 g)	F	10	
				M	20	
				NA	3	
Arrhenius breakpoint temperature test (f_{Hmax}, T_{AB}, T_{PEAK}, T_{ARR}, $PEAK_{fHmax}$) and Ventricle mass	wild	No	Adult (> 50 g)	F	7	30
				M	5	
		No	Juvenile (< 50 g)	F	4	
				M	5	
				NA	4	
	lab	No		F	1	
				M	3	
				NA	1	

Supplemental Figures and Figure legends

Figure S1.

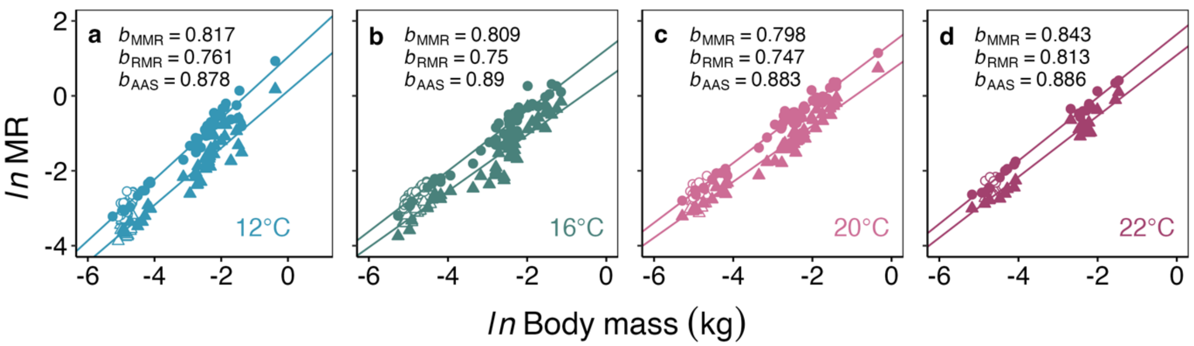

Figure S1. Temperature-specific hypometric scaling of maximum and resting metabolic rates across acute temperatures in barred surfperch. (**a,b,c,d**) Temperature specific scaling relationships of MMR (circles) and RMR (diamonds) varied leading to higher scaling of absolute aerobic scopes (AAS), which can be visualized as the "gap" between the plotted MMR and RMR slopes. The open symbols show laboratory-born juveniles. Fish were repeat tested at each temperature; MMR: n = 83 (238); RMR: n = 81 (233), individuals (observations).

Figure S2.

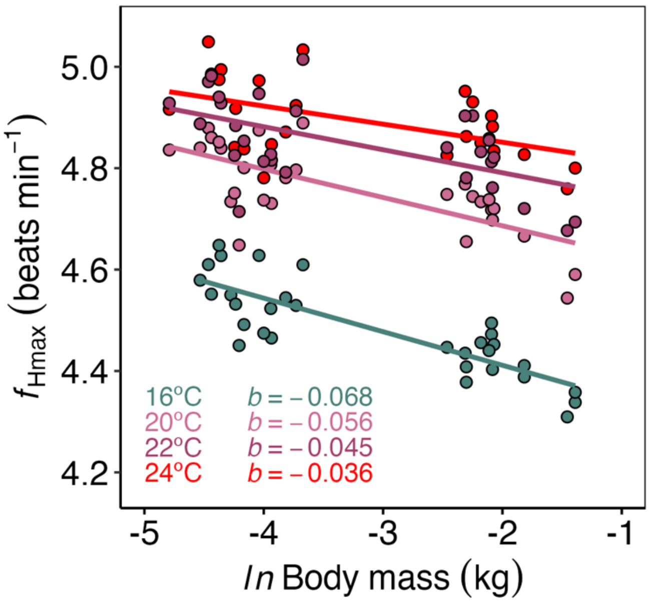

Figure S2. Negative temperature-specific scaling of maximum heart rate (f_{Hmax}) in barred surfperch across acute temperatures.

Individual f_{Hmax} was measured across acutely increased temperatures. Plotted are scaling relationships at each acute temperature separately (16, 20, 22, and 24ºC; simple linear models).

Figure S3.

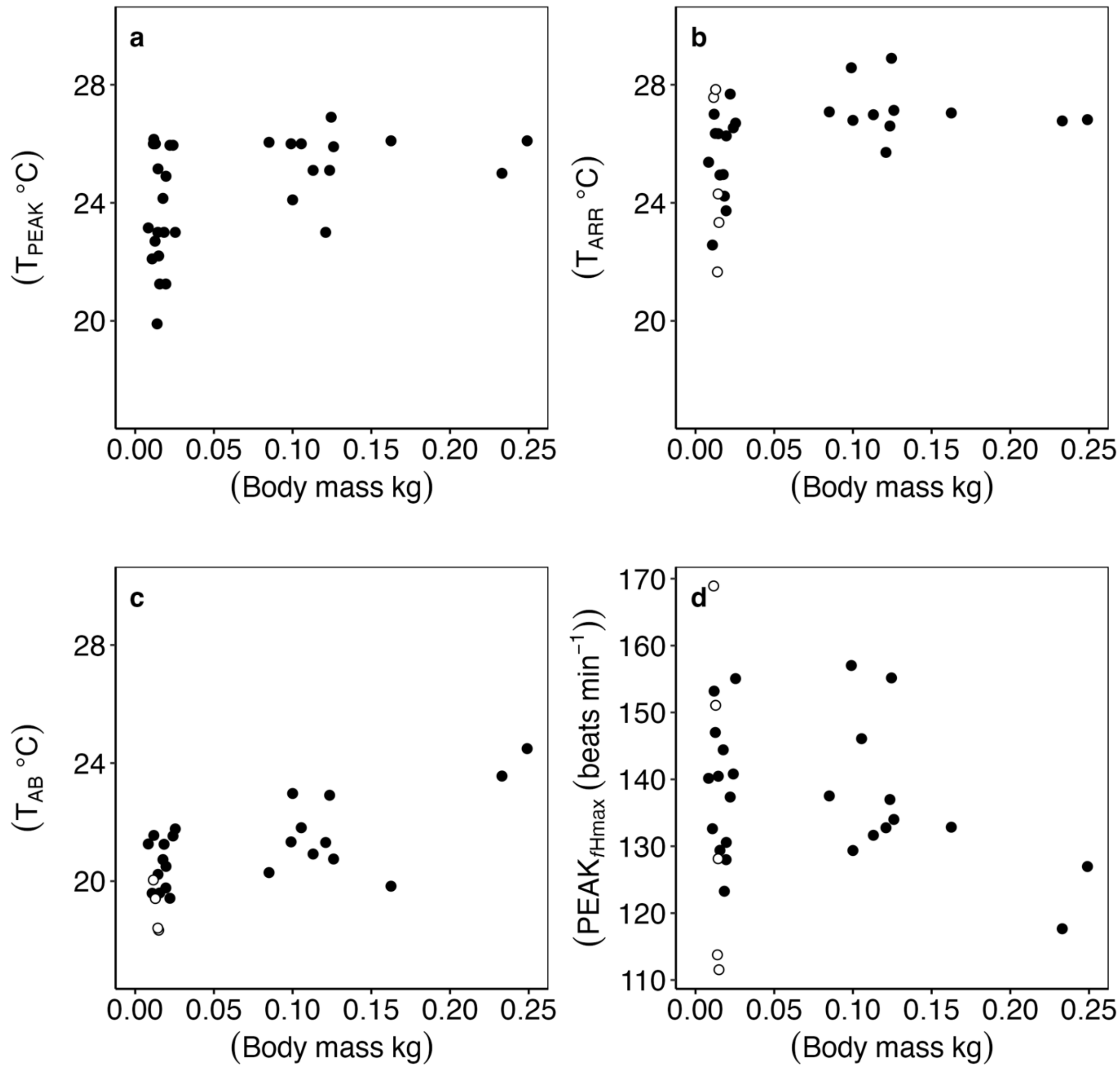

Figure S3. Mass scaling of cardiac thermal performance indices.
Plotted are: T_{PEAK} (**a**), T_{ARR} (**b**), T_{AB} (**c**), PEAK f_{Hmax} (**d**). In T_{AB} (**C**) the origin of individual — laboratory-born (open symbols) and wild-collected fish (closed symbols) — was a significant explanatory variable. The peak maximum heart rate at any temperature ($PEAK_{fHmax}$). (**d**) was not significantly explained by body mass. Size range: 8.3 to 249 g.

Figure S4.

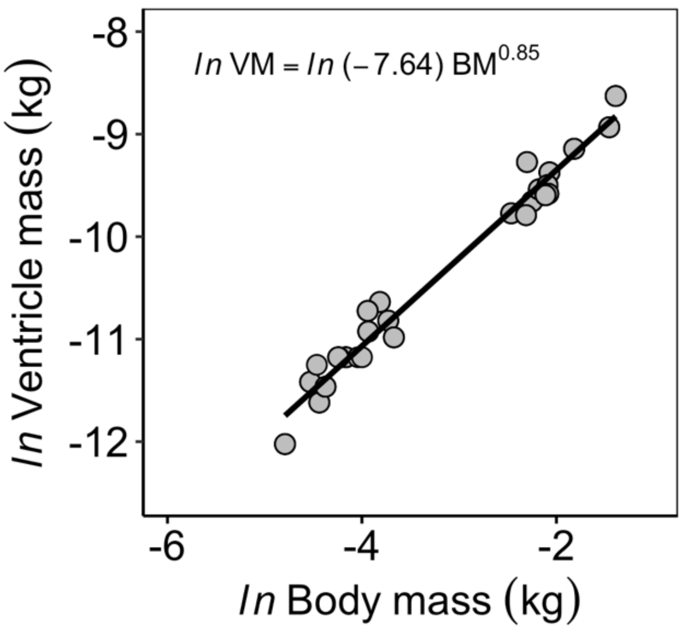

Figure S4. Scaling of ventricular mass.
Plotted are individuals used in the Arrhenius breakpoint temperature tests (n = 23). VM = ventricular mass.

Figure S5.

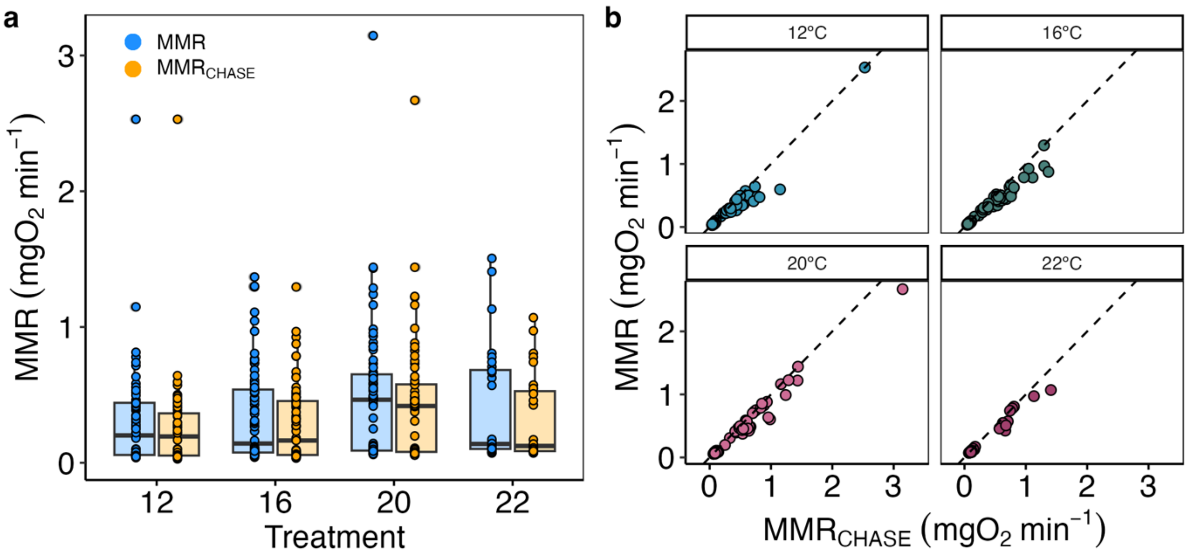

Figure S5. Comparison of individual maximum metabolic rate measurements. MMR$_{CHASE}$ is maximum metabolic rate measurement recorded following a 3-min chase and 1-min air exposure. MMR is the highest reported metabolic rate value observed at any time during the overnight respirometry trial. If MMR$_{CHASE}$ was the highest value, then MMR and MMR-chase are equivalent. (**a**) Boxplot showing MMR values of each individual at each acute temperature treatment. The MMR values represent maximum metabolic rates for the individual (mgO_2 min^{-1}). (**b**) Correlations between individual-specific MMR and MMR$_{CHASE}$ at each temperature treatment (panels; ºC noted on the top of each panel); the dashed lines are the "identity line" with slope = 1 and intercept = 0. The MMR$_{CHASE}$ and MMR are not statistically different, therefore using MMR measured overnight does not change the biological outcomes and conclusions.
